# Polyp size of 1 cm is insufficient to discriminate neoplastic and non-neoplastic gallbladder polyps

**DOI:** 10.1007/s00464-018-6444-1

**Published:** 2018-09-10

**Authors:** Sarah Z. Wennmacker, Aafke H. van Dijk, Joris H. J. Raessens, Cornelis J. H. M. van Laarhoven, Joost P. H. Drenth, Philip R. de Reuver, Iris D. Nagtegaal

**Affiliations:** 1 Department of Surgery, Radboud University Medical Centre, r 618, PO Box 9101, 6500 HB Nijmegen, The Netherlands; 2 Department of Surgery, Academic Medical Centre, Amsterdam, The Netherlands; 3 Department of Gastroenterology and Hepatology, Radboud University Medical Centre, Nijmegen, The Netherlands; 4 Department of Pathology, Radboud University Medical Centre, Nijmegen, The Netherlands

**Keywords:** Gallbladder polyp, Cholecystectomy, Surgical threshold, Neoplasia

## Abstract

**Background:**

A significant proportion of gallbladder polyps are non-neoplastic, for which resection is not necessary. However, international guidelines advocate cholecystectomy for all polyps ≥ 1 cm. This study assessed a national cohort of histopathologically proven gallbladder polyps to distinguish neoplastic from non-neoplastic polyps.

**Methods:**

PALGA, the nationwide network and registry of histo- and cytopathology, was searched to identify all histopathologically proven gallbladder polyps between 2003 and 2013. All polyps and (focal) wall thickenings > 5 mm were included, and classified as neoplastic or non-neoplastic. Polyp subtype, size, distribution, presentation as wall thickening or protruding polyp, and presence of gallstones were assessed for neoplastic and non-neoplastic polyps. A decision tree to distinguish neoplastic and non-neoplastic polyps was made and diagnostic accuracy of 1 cm surgical threshold was calculated.

**Results:**

A total of 2085 out of 220,612 cholecystectomies contained a polyp (0.9%). Of these polyps, 56.4% were neoplastic (40.1% premalignant, 59.9% malignant) and 43.6% non-neoplastic (41.5% cholesterol polyp, 37.0% adenomyomatosis, 21.5% other). Polyp size, distribution, and presence of gallstones were reported in 1059, 1739 and 1143 pathology reports, respectively. Neoplastic polyps differed from non-neoplastic polyps in size (18.1 mm vs 7.5 mm, *p* < 0.001), singularity (88.2% vs 68.2%, *p* < 0.001), wall thickening (29.1% vs 15.6%, *p* < 0.001), and presence of gallstones (50.1% vs 40.4%, *p* = 0.001). However, adenomyomatosis presented with similar characteristics as neoplastic polyps. Fifty percent of polyps were ≥ 1 cm surgical threshold (optimal surgical threshold based on ROC-curve); sensitivity for indicating neoplastic polyps was 68.1%, specificity was 70.2%, and positive and negative predictive values were 72.9% and 65.1%.

**Conclusions:**

The prevalence of gallbladder polyps on cholecystectomy is low and many of the polyps are non-neoplastic. Clinicopathological characteristics differ between neoplastic and non-neoplastic polyps in general, but these cannot properly indicate neoplasia. The 1 cm surgical threshold has moderate diagnostic accuracy and is insufficient to indicate surgery for neoplastic gallbladder polyps.

Annually, 800,000 cholecystectomies are performed in the United States and 23,000 in the Netherlands [1, 2]. Gallbladder polyps, defined as elevated (mucosal) lesions projecting into the lumen of the gallbladder [3], are found in 0.6–4% of cholecystectomies [4–6]. Gallbladder polyps can be categorized as neoplastic or non-neoplastic polyps based on histopathological evaluation. Neoplastic polyps include all cancerous lesions (most commonly adenocarcinoma) and precursors of cancer (all types of adenomas) [3, 7–9]. Non-neoplastic polyps consist of an aggregation of tumor-like lesions without malignant potential, including cholesterol polyps, inflammatory polyps, and adenomyomatosis [3, 7, 8]. An accurate estimate of the prevalence of neoplastic and non-neoplastic polyp types is lacking.

The presence of gallbladder polyps causes a clinical problem, since surgery is only absolutely indicated for neoplastic polyps, including adenomas in view of their assumed malignant potential [10–13]. While international guidelines have various recommendations for the indication of cholecystectomy in general [14], it is of interest that American [15] and other western countries [16–18] advocate cholecystectomy for polyps ≥ 1 cm. For polyps < 1 cm, cholecystectomy is only suggested in patients with additional risk factors for malignancy (e.g., older age or primary sclerosing cholangitis (PSC)) or in case of biliary symptoms without alternative causes [16, 17].

The surgical threshold size is based on older retrospective studies that demonstrated that polyps with a diameter ≥ 1 cm are more likely to be neoplastic [7, 19–22]. However, the accuracy of size as a determinant of malignant potential is limited [22–25]. Identification of clinicopathological or clinical characteristics that distinguishes gallbladder polyps on basis of the malignant potential is desirable. Discriminative characteristics could improve pre-operative work-up and optimize indication for cholecystectomy, to avoid unnecessary surgery related morbidity and costs.

In order to achieve this goal, we collected clinicopathological data over a 10-year period from all gallbladder polyps that were available from the Dutch nation-wide pathology registry. Our aim was to define characteristics that enable differentiation between neoplastic and non-neoplastic polyps.

## Methods

### Patient identification

We searched PALGA, the Dutch nationwide network and registry of histopathology and cytopathology, to identify all patients with histopathologically proven gallbladder polyps between January 2003 and December 2013 in the Netherlands. PALGA contains pathology reports of all pathology laboratories of academic and non-academic hospitals in the Netherlands, and has complete coverage of reports since 1991 [26]. A search was performed with the search term “gallbladder” combined with “polyp” or “adenoma” or “adenomatous polyp” or “hyperplastic polyp” or “dysplasia” or “cholesterolosis” or “all benign neoplasms” or “all primary malignancies (incl. cis)”, or the individual search term “cholesterol polyp”. The search was restricted to histological samples of patients ≥ 18 years of age. Patients were further included or excluded after critical review of the individual pathology reports. Biopsies, cholecystectomies performed as part of primary non-gallbladder surgery (e.g., pancreatectomy or hepatectomy), and duplicates (e.g., due to second opinions at another pathology lab) were excluded. All patients with a polyp or (focal) wall thickening > 5 mm in the gallbladder, were included.

### Datacollection and histopathologicl assessment

The following variables were, if reported, extracted from the individual pathology reports: polyp subtype, polyp size, number of polyp(s), presentation as protruding polyp or wall thickening, and presence of gallstones. All polyps were subsequently categorized as neoplastic ((cyst)adenoma, adenocarcinoma, or other malignancies) or non-neoplastic (all other polyp subtypes). If both neoplastic and non-neoplastic polyps were present in one gallbladder, overall polyp type was categorized as neoplastic. If multiple histopathological subtypes of neoplastic polyps were present, overall subtype was classified according the most severe neoplastic histopathological subtype. If multiple histopathological subtypes of non-neoplastic polyps were present, overall subtype was classified according the most relevant (in presence or amount) non-neoplastic histopathological subtype. Polyp size was classified as the largest measurement from the pathology report, and in case of multiple polyps as the size of the largest polyp present.

### Outcomes and analyses

Prevalence of gallbladder polyps was defined as the proportion of patients presenting with gallbladder polyps per 100,000 primary cholecystectomies between 2003 and 2013. Clinicopathological characteristics (size in mm, number of polyps, presentation as wall thickening, and presence of gallstones) were reported for neoplastic and non-neoplastic polyps in general, and for main neoplastic and non-neoplastic subtypes. Number or patients for whom these characteristics were available will be reported. Chi square and independent student *T* test statistics were performed to compare groups. A *p* value < 0.05 was considered statistically significant. Proportions of the different subgroups above and below surgical threshold of 1 cm were reported separately. Sensitivity, specificity, and predictive values of the 1 cm threshold were calculated, and a receiver operating characteristic (ROC) curve was used to determine the optimal size threshold for differentiating neoplastic and non-neoplastic polyps. The surgical threshold data and clinicopathological characteristics were assessed in a simple decision tree to establish combined discernment for neoplastic and non-neoplastic polyps. All statistical analyses were performed using SPSS Statistics 22.0 (IBM, Amsterdam, The Netherlands). All authors had access to the study data and have reviewed and approved the final manuscript.

## Results

### Epidemiology

**Fig. 1:**
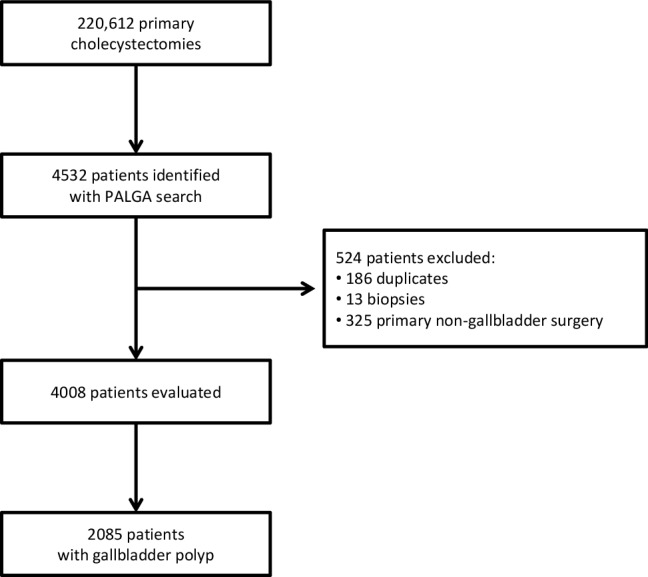
Flowchart patient identification

We identified a total of 220,612 primary cholecystectomies between 2003 and 2013. The search strategy resulted in 4008 evaluated patients, and subsequent critical review of the pathology reports identified 2085 (0.9%) patients with a gallbladder polyp (Fig. 1). The prevalence of gallbladder polyps was 945 per 100,000 cholecystectomies.

**Fig. 2:**
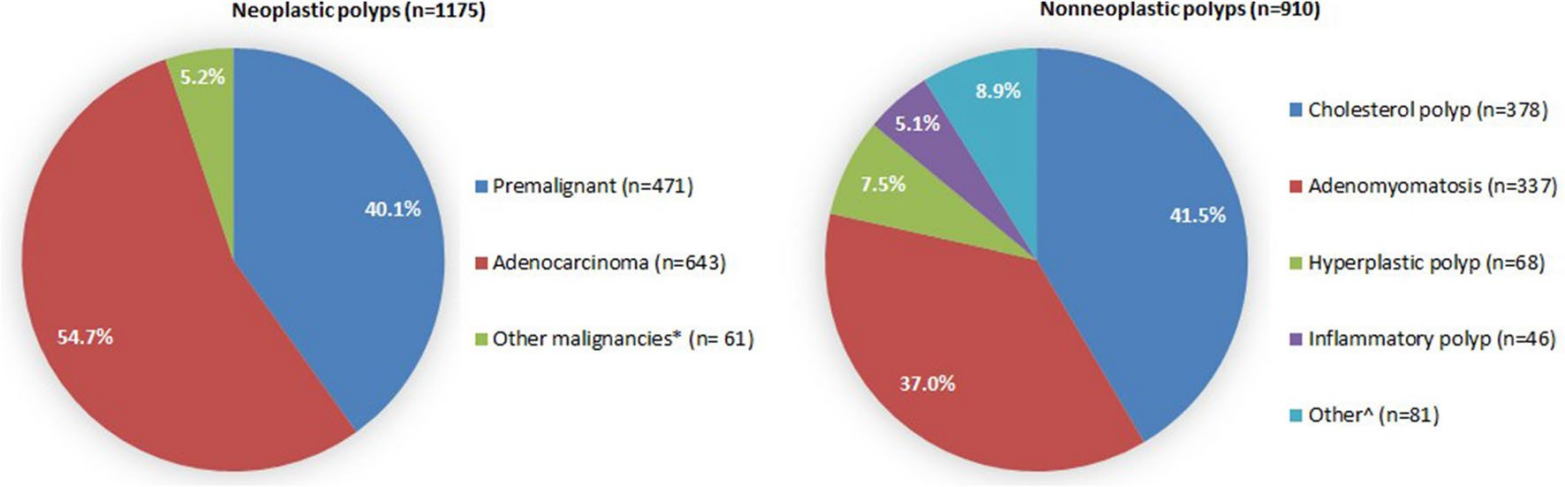
Histopathological subtypes of neoplastic and non-neoplastic polyps. *Consists of: squamous cell carcinoma (*n* = 13), lymphoma (*n* = 10), metastasis (*n* = 9), carcinoïd (*n* = 5), other neuroendocrine tumor (*n* = 5), sarcoma (*n* = 5), papillary carcinoma (*n* = 4), undifferentiated carcinoma (*n* = 4), granular cell myoblastoma (*n* = 2), melanoma (*n* = 1), colloid carcinoma (*n* = 1), spindle and giant cell carcinoma (*n* = 1), small cell carcinoma (*n* = 1). ^Consists of: hemangioma (*n* = 4), lipoma (*n* = 3), lymphangioma (*n* = 3), fibroma (*n* = 3), follicular cholecystitis (*n* = 2), and non-specified polyp (*n* = 66)

Eleven hundred seventy-five polyps (56.4%) were neoplastic and 910 (43.6%) were non-neoplastic. Neoplastic polyps consisted of 471 (40.1%) premalignant and 704 (59.9%) malignant polyps. The main non-neoplastic subtypes were cholesterol polyps (*n* = 378, 41.5%) and adenomyomatoses (*n* = 337, 37.0%). The histopathological subtypes of neoplastic polyps and non-neoplastic polyps are illustrated in Fig. 2.

### Clinicopathological characteristics

Polyp size, number of polyps, and presence of gallstones were respectively reported in 1059, 1739 and 1143 pathology reports. Information on presentation as protruding polyp or wall thickening was available for all polyps. Neoplastic polyps were larger compared to non-neoplastic polyps (18.1 mm vs 7.5 mm, *p* < 0.001) and more often were present as a single polyps (88.2% vs 68.2%, *p* < 0.001), as wall thickening rather than a lumen protruding polyp (29.1% vs 15.6%, *p* < 0.001), and in the presence of gallstones (50.1% vs 40.4%, *p* = 0.001). There were a number of characteristics that were present more often in malignant neoplastic polyps compared to premalignant neoplastic polyps such as a larger size (23.1 mm vs 10.9 mm, *p* < 0.001), presentation as a single polyp (95.2% vs 76.0%, *p* < 0.001), and presentation as wall thickening (37.9% vs 15.9%, *p* < 0.001) (Table 1).

**Table 1:** Clinicopathological characteristics of neoplastic and non-neoplastic polyps

	Size in mm (*n* = 1059), *µ* (SD)^#1^	Single polyp (*n* = 1739), *n*/*n* (%)	Wall thickening (*n* = 2085), *n*/*n* (%)	Gallstones (*n* = 1143), *n*/*n* (%)
*Neoplastic*	18.1 (17.9)*	847/960 (88.2)*	342/1175 (29.1)*	318/635 (50.1)*
Adenoma (incl. dysplasia)	10.9 (8.8)^^^	266/350 (76.0)^	75/471 (15.9)^^^	144/294 (49.0)
Malignant polyp	23.1 (20.6)	581/610 (95.2)	267/704 (37.9)	174/341 (51.0)
*Non-neoplastic*	7.5 (5.9)	531/779 (68.2)	142/910 (15.6)	205/508 (40.4)
Cholesterol polyp	5.2 (5.1)^+^	138/327 (42.2)^+^	3/378 (0.8)^+^	70/221 (31.7)^+^
Adenomyomatosis	10.8 (5.7)^+^	280/291 (96.2)^+^	111/337 (32.9)^+^	92/187 (49.2)

*Significantly different from nonneoplastic polyps (all *p* < 0.001); ^significantly different from malignant polyps (all *p* < 0.001); ^+^significantly different from other nonneoplastic polyps (all *p* < 0.003)

Clinicopathological characteristics also differed within main non-neoplastic subtypes, as illustrated in Table 1. Interestingly, adenomyomatosis differed from other non-neoplastic polyps on all assessed characteristics, but presented with characteristics similar to neoplastic polyps in general (presentation as wall thickening and in the presence of gallstones), premalignant polyps (size), or malignant polyps (number of polyps).

### Surgical threshold

**Fig. 3:**
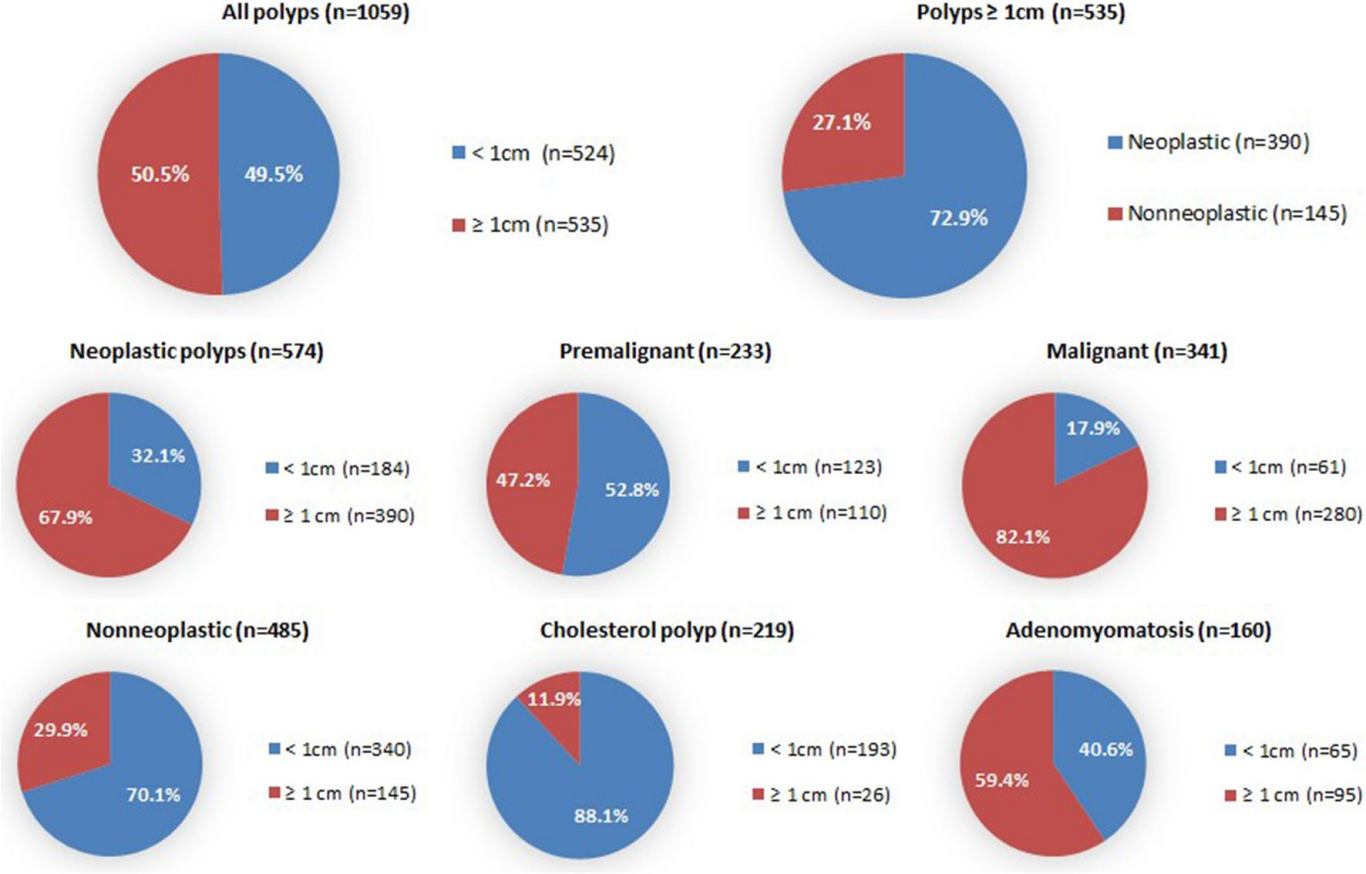
Surgical threshold. Gallbladder polyps and subgroups above and below surgical threshold of 1 cm. Neoplastic polyps significantly differed from non-neoplastic polyps (*p* < 0.001). Pre-malignant polyps significantly differed from malignant polyps (*p* < 0.001). Cholesterol polyps significantly differed from other non-neoplastic polyps (*p* < 0.001). Adenomyomatosis significantly differed from other non-neoplastic polyps (*p* < 0.001)

**Fig. 4:**
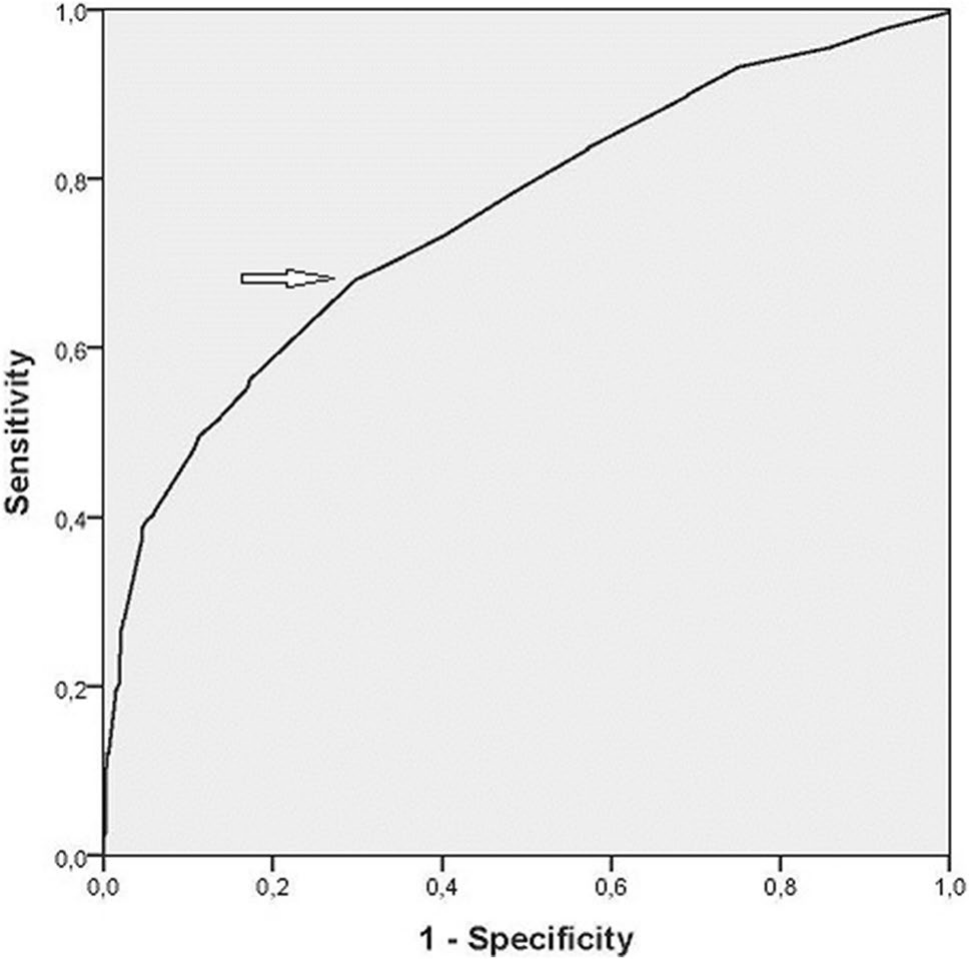
ROC-curve polyp size. ROC-curve of polyp size compared for neoplastic polyp type. Area under the curve: 0.75 (95% CI 0.72–0.78). Optimal diagnostic cut-off size is 1 cm with sensitivity of 0.68 and specificity of 0.70 (*p* < 0.001). Sensitivities and specificities for all cut-off values are provided as supplementary table

Fifty percent (*n* = 535) of the polyps, for which size was available, met the ≥ 1 cm surgical threshold; 67.9% of neoplastic polyps and 29.9% of non-neoplastic polyps (*p* < 0.001) (Fig. 3). The ROC-curve (Fig. 4) showed that 1 cm is the most optimal size threshold for differentiating neoplastic and non-neoplastic polyps. Sensitivity of the surgical threshold for indicating neoplastic polyps was 68.1%, specificity was 70.2%, and positive and negative predictive values were 72.9% and 65.1%. The 1 cm surgical threshold would have identified 47.2% of pre-malignant and 82.1% of malignant polyps, and 11.9% of cholesterol polyps and 59.4% of adenomyomatoses.

### Prediction of neoplastic and non-neoplastic polyp type

**Fig. 5:**
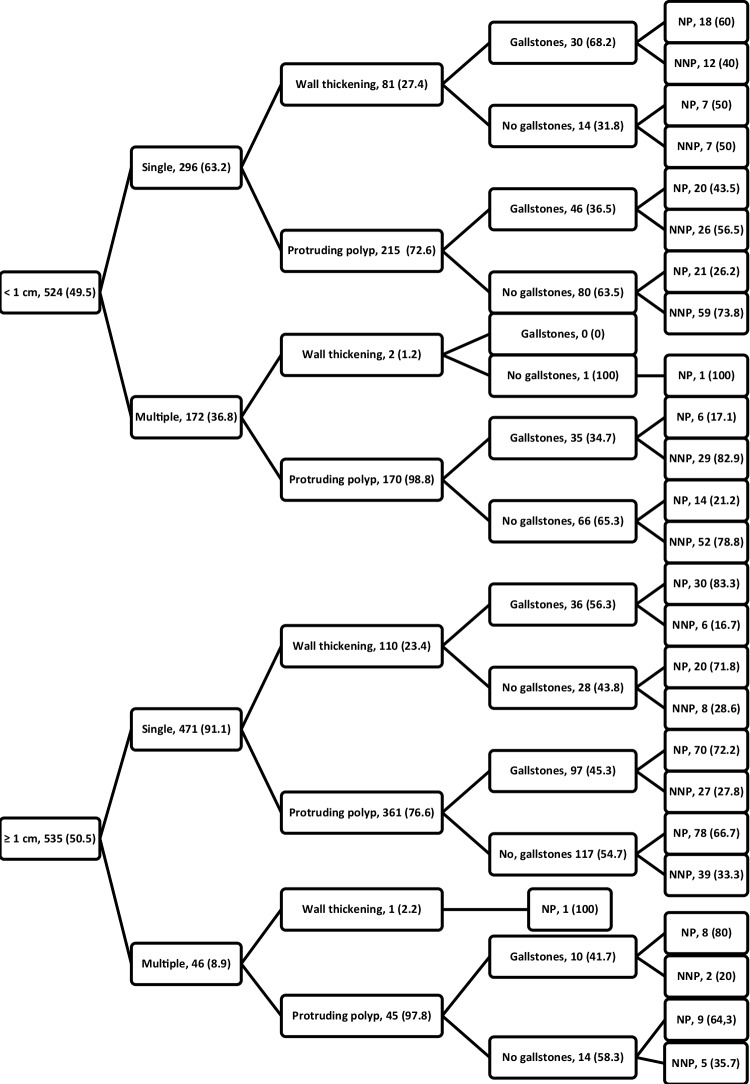
Surgical threshold and clinicopathological characteristic decision tree. Presented as characteristic, *n* (%). Size available for *n* = 1059, number of polyps and wall thickening for *n* = 985, presence of gallstones and polyp type *n* = 574. *NP* neoplastic polyp, *NNP* nonneoplastic polyp

We established the decision tree in Fig. 5 using the surgical threshold data and clinicopathological characteristics of neoplastic and non-neoplastic polyps. The model starts with polyp size under or above 1 cm, followed by branches indicating number of polyps, presentation, and presence of gallstones. This decision tree results in the prediction of neoplastic or non-neoplastic polyp type for each of the 16 possible combinations of clinicopathological characteristics. The highest chances of neoplasia are seen for single polyps ≥ 1 cm presenting as wall thickening in the presence of gallstones, and for multiple polyps presenting as wall thickening irrespective of size and gallstones (83.3% and 100% respectively). Multiple protruding polyps < 1 cm in the presence of gallstones have the highest chance of being non-neoplastic polyps (82.9%). The lowest predictive combination is for single polyps < 1 cm presenting as wall thickening without gallstones (50% chance of neoplastic c.q. non-neoplastic polyp).

## Discussion

This large nation-wide cohort study provides epidemiological and clinicopathological data of gallbladder polyps over a 10-year period in the Netherlands. The present study illustrates that neoplastic gallbladder polyps generally differ from non-neoplastic gallbladder polyps in size, number, presentation, and concomitant gallstones, but not from adenomyomatosis subtype. Ultimately, this study shows the moderate diagnostic accuracy of the current size based surgical threshold for cholecystectomy in gallbladder polyps. Even though 1 cm is the most optimal size threshold for differentiating neoplastic and non-neoplastic polyps, it would indicate incorrect treatment for nearly a third of the patients with gallbladder polyps.

The relatively low prevalence of gallbladder polyps on cholecystectomy established in this study is in line with international studies [4–6]. Almost half of the polyps found on histopathological examination after cholecystectomy were non-neoplastic. Although common consensus is that only symptomatic or potentially malignant polyps need surgery [16, 17], it remains difficult to properly differentiate between neoplastic and non-neoplastic polyps during the pre-operative phase. This study sought to identify clinicopathological characteristics associated with neoplastic nature of gallbladder polyps, in order to improve decision making in the pre-operative setting.

In concordance with previous studies [27–29], we confirm that large polyp size, presentation as a single polyp, presentation as wall thickening, and presence of gallstones, were all generally more frequent in neoplastic polyps than non-neoplastic polyps. Neoplastic polyps also significantly more often exceeded the 1 cm threshold. Superficially, these results are in support of international guidelines, which advocate cholecystectomy for polyps ≥ 1 cm [16–18, 30]. Recently, the accuracy of this surgical threshold as indicator for surgery has been doubted [31, 32]. Even though the clincopathological differences in our study are statistical significant, we underline that neither size nor simple clinicopathological characteristics are sufficient to truly indicate neoplastic polyps for cholecystectomy.

The ROC-curve in our study confirms that 1 cm is the most optimal size threshold for differentiating neoplastic and non-neoplastic polyps, and thus for patient selection for cholecystectomy. However, based on the 1 cm threshold alone, still 32% of patients with neoplastic polyps (18% of gallbladder cancers and 53% of precursor lesions) would be withheld from surgery. Additionally, almost 900 unnecessary cholecystectomies for non-neoplastic polyps would be performed in the United States every year. A treatment threshold with a misclassification rate of nearly one in three patients is precarious to use in daily clinical practice. Additional patient characteristics, such as risk factors for malignancy (e.g., age, PSC, Indian ethnicity) or biliary symptoms [16, 17] that are evaluated at a second stage, may indicate some of the patients with neoplastic polyps < 1 cm for surgery. However, as long as the primary selection in all guidelines is polyp size [16–18, 30], mainly asymptomatic patients with non-neoplastic polyps ≥ 1 cm continue to be unnecessarily operated.

Increase or decrease of the surgical threshold alone will not improve patient selection for cholecystectomy. A size threshold of > 15 mm would exclude nearly all non-neoplastic polyps for cholecystectomy, but up to 60% of neoplastic polyps would be missed. A lower cut-off of for example 6 mm, as suggested by Zielinski et al. [24], would only increase sensitivity of the surgical threshold to 78%, but would drop specificity to 52%. Combination of polyp size with other polyp characteristics assessed in this study, could only improve patient selection for cholecystectomy in specific scenarios. Five out of 16 possible clinicopathological combinations had an 80% or higher prediction of polyp type. Other scenarios were as good as the 1 cm threshold alone, or even dropped to a 50–50 change of neoplastic or non-neoplastic polyp type.

Pathological and radiological characteristics are equally important in the differentiation of gallbladder polyps. Although pre-operative patient selection is based on radiological imaging, it visualizes a (clinico)pathological feature of the polyp. If the pathological substrate that is being visualized is not distinctive, the subsequent radiological image will not be either. The first step towards optimized patient selection for cholecystectomy is identification of neoplastic and non-neoplastic histopathological subtypes. This study showed that adenomyomatosis, accounting for over one-third of non-neoplastic polyps, has more clinicopathological similarities with neoplastic polyps, than with other non-neoplastic subtypes. Therefore, general polyp classification as “neoplastic” or “nonneoplastic” is not useful to aid in clinical decision making. A recent study suggested classification of polyps into three categories: “cholesterol”, “benign non-cholesterol” and “malignant polyps”, placing all non-neoplastic polyps other than cholesterol polyps in the same category as adenomas [31]. We strongly suggest differentiating more distinct subgroups, since adenomas and the non-neoplastic subtypes require contrary treatment. Previous studies suggested that detailed polyp features, including polyp shape, surface, and radiological internal echogenic patterns should be able to differentiate between cholesterol polyps, inflammatory polyps, adenomyomatosis, adenomas, and malignant polyps [33–35]. Diagnostic accuracy of this dedicated (endoscopic) ultrasound imaging and the value of additional modalities, such as MRI, should be (re)assessed.

The strengths of this study are the large nationwide study population and full coverage of all pathology reports on gallbladder polyps in a 10-year period in the Netherlands. PALGA contains pathology reports of all Dutch pathology labs, both academic and non-academic. Pathology reports can be provided until the end of the year following the year the histopathology analysis was conducted. At time of data collection, PALGA database was fully updated up to and including 2013. Therefore, the presented data fully covered all primary cholecystectomies performed for gallbladder polyps in a 10-year period in the Netherlands.

Limitations of this study include the incomplete information on clinicopathological characteristics from the pathology reports and lack of clinical and radiological characteristics of the included patients. A specified decision tree for distinct neoplastic and non-neoplastic subtype could not be provided. PALGA only collects histopathological data, without indicating whether these cholecystectomies were performed because of the gallbladder polyps, or that the polyps were incidental findings. This may have caused the inclusion of more small polyps, that were below the threshold of pre-operative clinical evaluation. Additionally, we included focal wall thickenings > 5 mm as polyps under the hypothesis that some histological subtypes (malignancies, adenomyomatosis and inflammatory polyps) have the tendency to present as focal wall thickening or sessile lesions, rather than protruding polyp [33, 34, 36]. We chose the cut-off of 5 mm for focal wall thickening, as gallbladder walls up to 5 mm can be normal (e.g., due to contraction of the gallbladder) [37]. Focal gallbladder wall thickenings > 5 mm will be classified as aberrant on US and are subsequently relevant to the clinicians. However, our cohort may have been biased towards a relative higher percentage of these specific polyp types and smaller polyps.

In conclusion, this large nationwide cohort study establishes that ~ 1% of cholecystectomies contain a polyp and that 56% of these polyps are neoplastic. Polyp size, number of polyps, presentation as wall thickening and presence of gallstones differ between neoplastic and non-neoplastic polyps, but cannot properly identify neoplastic polyps. Even though polyp size of 1 cm is the best available size based surgical threshold, it is insufficient to indicate cholecystectomy for gallbladder polyps, due to its moderate diagnostic accuracy.
